# Spatial morphological and molecular differences within solid tumors may contribute to the failure of vascular disruptive agent treatments

**DOI:** 10.1186/1471-2407-12-522

**Published:** 2012-11-15

**Authors:** Linh Nguyen, Theodora Fifis, Caterina Malcontenti-Wilson, Lie Sam Chan, Patricia Nunes Luiza Costa, Mehrdad Nikfarjam, Vijayaragavan Muralidharan, Christopher Christophi

**Affiliations:** 1 Department of Surgery, University of Melbourne, Austin Health, Heidelberg, Victoria, 3084, Australia

**Keywords:** Vascular disruptive agent, OXi4503, Tumor periphery, Hypoxia, Growth factor, Infiltrating cells, EMT

## Abstract

**Background:**

Treatment of solid tumors with vascular disrupting agent OXi4503 results in over 90% tumor destruction. However, a thin rim of viable cells persists in the tumor periphery following treatment, contributing to subsequent recurrence. This study investigates inherent differences in the microenvironment of the tumor periphery that contribute to treatment resistance.

**Methods:**

Using a murine colorectal liver metastases model, spatial morphological and molecular differences within the periphery and the center of the tumor that may account for differences in resistance to OXi4503 treatment were investigated. H&E staining and immunostaining were used to examine vessel maturity and stability, hypoxia and HIF1α levels, accumulation of immune cells, expression of proangiogenic factors/receptors (VEGF, TGF-β, b-FGF, and AT1R) and expression of EMT markers (ZEB1, vimentin, E-cadherin and β-catenin) in the periphery and center of established tumors. The effects of OXi4503 on tumor vessels and cell kinetics were also investigated.

**Results:**

Significant differences were found between tumor periphery and central regions, including association of the periphery with mature vessels, higher accumulation of immune cells, increased growth factor expression, minimal levels of hypoxia and increased evidence of EMT. OXi4503 treatment resulted in collapse of vessels in the tumor center; however vasculature in the periphery remained patent. Similarly, tumor apoptosis and proliferation were differentially modulated between centre and periphery after treatment.

**Conclusions:**

The molecular and morphological differences between tumor periphery and center may account for the observed differential resistance to OXi4503 treatment and could provide targets for drug development to totally eliminate metastases.

## Background

Solid tumors require a well established vasculature to grow. As the tumor grows its vasculature undergoes constant remodeling [1] which makes the tumor microvasculature unstable. This characteristic makes the tumor microvasculature more sensitive to destabilizing drugs compared to normal host microvasculature. Exploiting these differences to target established tumor microvasculature is a novel concept resulting in the development of vascular disruptive agents (VDAs) [2]. Treatment with VDAs is characterized by rapid and extensive destruction of tumor limited only by the persistence of a viable rim of tumor in the periphery which subsequently leads to recurrence [3]. The Combretastatins are a family of tubulin binding vascular disrupting agents that specifically target the vascular network within a solid tumor. Despite extensive tumor destruction, complete tumor eradication is not achieved [4]. OXi4503, a derivative of Combretastatin CA4P, is a second generation VDA that is more potent than CA4P, killing more than 90% of tumor [5]. It has been shown to be effective in a wide variety of tumor models and is currently undergoing clinical trials (ClinicalTrials.gov Identifier: NCT01085656). Despite its enhanced potency, treatment with OXi4503 also leaves the characteristic rim of viable tumor cells albeit smaller in size than that seen in tumors treated with CA4P [6,7].

As tumor cells survive only in the periphery, we hypothesize that there are intrinsic differences between the periphery and the bulk of the tumor that confer resistance to treatment. A number of studies reported increased expression of growth factors in the periphery [8,9]. In a previous study [10] we have shown that macrophages and T-cells infiltrate the tumor and preferentially accumulate in the periphery. Other studies indicate that tumor associated immune cells secrete cytokines and growth factors that promote tumor growth [11-14].

The present study examines inherent differences between the periphery and the bulk of the tumor in a murine model of colorectal liver metastases including vessel morphology, immune cell infiltration, expression of pro-angiogenic factors and markers of Epithelial to Mesenchymal Transition (EMT). Morphological and molecular changes occurring in the tumor vasculature and in tumor cell kinetics following administration of OXi4503 are also investigated.

## Methods

### Animals

Six to eight week old male CBA mice (Laboratory Animal services, University of Adelaide, South Australia) were used in all experiments. Mice were maintained in standard cages with access to irradiated food and water ad libitum, and exposed to a twelve hour light/dark cycle. All procedures were implemented in accordance with the guidelines of the Austin Health Animal Ethics Committee.

### Experimental model of colorectal cancer liver metastases (CRCLM)

The primary cancer cell line MoCR was derived from a dimethyl hydrazine (DMH)-induced primary colon carcinoma in the CBA mouse and maintained *in vivo* by serial passage in the flanks of CBA mice [15]. For passage and experimentation, subcutaneous tumors were teased, passed through a filter, treated with EDTA and washed in PBS to make a single cell suspension. Liver metastases were induced by intrasplenic injection of 5x10^4^ tumor cells prior to splenectomy as reported previously [15]. In this model, liver metastases are fully established by 21 days following tumor induction. The tumor morphology and growth patterns in this model have been described previously [6,15,16]. Metastases of varying sizes are found throughout the liver. The metastasis pattern is very similar and reproducible within a group of mice. The whole liver is sliced in sections of 2 mm thickness. Cross-sections of the larger tumors are represented in more than one section. Random sections are selected to represent the entire liver and used for paraffin embedding and analysis. Each section could contain from one to several individual tumors (Additional file 1: Figure S1). Metastases seeded in close proximity often coalesce into a continuous tumor.

### Treatment protocol

Treatment was administered sixteen days after induction of liver metastases when tumors are well established. OXi4503, kindly donated by OXiGENE (OXiGENE® Inc. South San Francisco, CA), was freshly prepared by dissolving in 0.9% sterile saline (NaCl) and protected from light. A single maximum tolerated dose of OXi4503, determined previously to be 100 mg/kg [16], was administered via intraperitoneal injection. Control groups were administered an equivalent volume of sterile saline. Tissues were collected at one hour, twenty four hours and five days following OXi4503 treatment.

### Definition of tumor periphery

Tumor periphery in our studies consisted of the area covering the tumor-host interface and extending one hundred microns towards the tumor center. All the remaining tumor area was considered part of the tumor center.

### Vascular morphology

Vessel morphology was examined microscopically in stained tumor sections. Immature vessels and/or vessels undergoing angiogenesis were detected by CD34 staining [17]. All CD34 positive vessels/mm^2^ in each tumor section were counted. Vessel stability and maturity were also assessed by pericyte coverage and angiopoetin 1(Ang1) association [18]. The presence of pericytes was visualised by αSMA immunostaining and enumerated by counting of αSMA positive tumor vessels in serial sections stained for αSMA or CD34. Only vessels that stained for both markers were included in the enumeration. Ang1 association was determined by double immunostaining for Ang1 and CD34.

### Detection of tumor hypoxia

Pimonidazole was used as a marker of tumor hypoxia. Pimonidazole hydrochloride was dissolved into 0.9% NaCl and administered intravenously to tumor-bearing mice in doses of 30 mg/kg. The livers were removed one hour after pimonidazole administration and fixed in 10% formalin in 0.1M phosphate buffer, pH 7.2. Hypoxic tumor regions were detected immunohistochemically as reported previously [19].

### Assessment of epithelial to mesenchymal transition (EMT)

The main indicators of EMT are down regulation of the cell junction protein E-cadherin, nuclear accumulation of β-catenin another junctional protein, up regulation of the mesenchymal marker vimentin and up regulation of transcription inhibitors of epithelial proteins such as ZEB1 [20,21]. The spatial expression of these markers was assessed for evidence of EMT.

### Histological assessment

Hematoxylin and eosin (H & E) stained sections were examined histologically and digital images captured using a Nikon Coolscope® (Nikon Corporation, Chiyokd-ku, Tokyo, Japan). A minimum of 50 tumors were assessed per treatment group.

### Immunohistochemistry

Spatial differences in untreated tumors and changes due to OXi4503 treatment were detected using histological and immunohistochemical techniques.

Antibodies used for infiltrating immune cells; Rabbit polyclonal antibodies to human CD3 (A0452, DAKO), Rat anti-mouse monoclonal antibodies to FOXP3 (14-5773-80, e-bioscience), and F4/80 a kind gift from Professor Mauro Sandrin Dept. of Surgery, University of Melbourne. Antibodies used for growth factor detection; Rabbit polyclonal antibodies to mouse AT1R (sc-1173), TGF-β (sc7892), b-FGF (Lot no: 24030710) obtained from Santa Cruz, VEGF (PC315, CalBiochem) and HIF1α (AB 3883, Chemicon). Antibodies used for vessel detection; Rat anti-mouse monoclonal antibodies to CD34 (MCA18256, Serotec), rabbit polyclonal antibodies to mouse CD31 (ab 28364, Abcam), αSMA (CME 305 AB, Biocare) and Angiopoetin1 (ab 8451–200, Abcam). Antibodies used for EMT detection; Rabbit polyclonal antibodies to mouse E-cadherin (sc-7870), Vimentin (sc-5568), ZEB1 (sc-25388) and rat anti-mouse monoclonal antibodies to β-catenin (sc-7199) all obtained from Santa Cruz. Cell proliferation was detected with rabbit monoclonal antibodies to Ki67 (rm-9106-s1 thermo scientific) and cell apoptosis with rabbit polyclonal antibodies to Active Caspase-3 (AF835, R&D systems). Additional file 2: Table S1 presents a list of antibody concentrations and assay conditions used.

Formalin fixed paraffin tissue sections (4 μm) were used with an indirect peroxidase labeling technique (Envision Plus, DAKO, Australia). Following deparaffinization and rehydration, endogenous peroxidase activity was blocked with 3% H_2_O_2_ and non-specific binding inhibited with 10% normal goat serum (01–6201 Zymed Laboratories, USA) after which epitope retrieval was conducted (Additional file 2: Table S1). Sections were incubated with primary antibodies overnight at 4°C. Negative controls were incubated with the respective non immune antibody isotypes or non-immunized rabbit IgG (Santa Cruz, sc-2027) at the same concentration as the primary antibody. Sections treated with the rat antibodies were subsequently treated with a rabbit anti-rat IgG linker antibody before treatment with a polymer based detection kit containing goat anti-rabbit immunoglobulins (IgG) linked to horseradish peroxidase (HRP) (Envision Plus, Dako, Australia). Each incubation step was followed by two five minute washes with PBS + 0.05% Tween 20. Positive staining was visualized using diaminobenzidine (DAB) as a substrate. For double immunostaining Vulcan fast red (Applied Medical FR805H) was used to stain CD34. Slides were counterstained with Mayer’s haematoxylin.

A minimum of five mice were used per group and between 75 and 120 tumors were assessed for each timepoint/treatment group. Images of stained tumors were captured using a digital light microscope (Nikon Coolscope®, Nikon Corporation, Japan) at between 10x and 400x magnification. The images of tumor fields were captured to be representative of the entire tumor, using a raster pattern which allowed for fields captured to be random and not overlap. Between 10 and 30 fields per tumor (periphery and center) were assessed. The images were analyzed using Image-Pro plus (Version 5, Media Cybernetics, Perth Australia). The number of CD34 positively stained vessels per tumor area (mm^2^) to were counted provide a microvascular density index. Ki67, active caspase3, CD3, FOXP3 and F4/80 were assessed as the number of CD34 positive cells per area of tumor (20x magnification). Positively stained cells per image were marked and quantification was performed using Image-Pro plus (Version 5, Media Cybernetics, Perth Australia). Differences in hypoxia and the antigens (AT1R, VEGF, b-FGF, TGF-β, HIF1α, E-cadherin, Vimentin, β-catenin and ZEB1) were assessed by microscopic observation and representative images are presented.

Quantification of AT1R, VEGF and TGF-β was performed using a semi-quantitative analysis. Areas of interest were identified using a light microscope (Olympus BH2, Japan) at a magnification of 125x. The entire margin of tumor host interface and tumor center were examined. Scoring criteria was used to estimate the amount and intensity of staining seen in each sample. The grading system used was: as: 0: no staining 1: faint staining; 2: small amount or weak staining; 3: moderate staining; 4: abundant or strong staining; 5: Abundant or very strong staining. Means for each group were determined using the individual average scores from each animal in the group. For all counting and scoring researchers were blinded in regard to the experimental group.

### Statistical analysis

Quantified data is represented as the mean ± standard error of the mean. Statistical analysis was conducted using SPSS (Statistical Package for the Social Sciences,^TM^ version 10, Chicago, Illinois, USA) with normality testing and use of both parametric and non parametric analytical tests as appropriate. All statistical tests were two-sided and a P value of 0.05 or less was considered statistically significant.

## Results

### Spatial differences in tumor vessel density and vessel morphology

**Figure 1:**
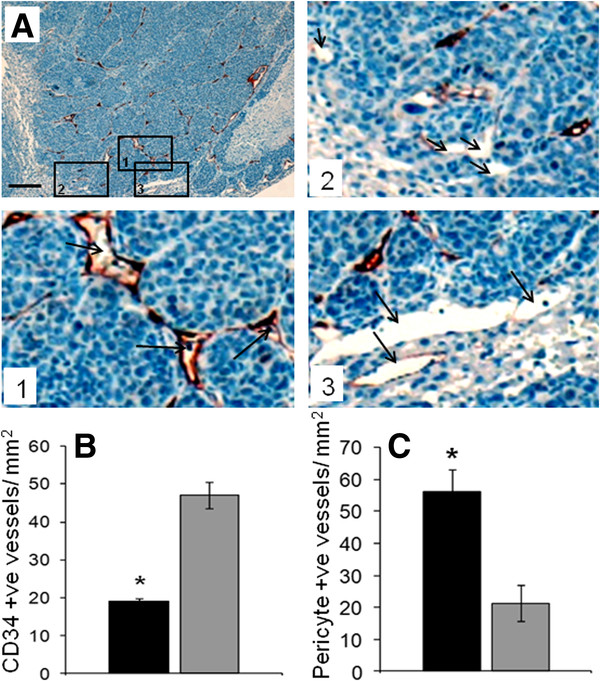
**Differences in blood vessel morphology between tumor periphery and center.A**: Formalin fixed liver sections with CRC liver metastases were stained with antibodies to CD34 (staining of endothelial cells on immature vessels). Scale bar=200μm. Inset 1 depicts tumor vessels (arrows) in the tumor center staining strongly for the CD34 endothelial marker. Inset 2 depicts tumor vessels (arrows) in the tumor periphery staining weakly for the CD34. Inset 3 depicts host vessels (arrows) being co-opted by the tumor displaying weak or no CD34 staining. **B**: Quantification of CD34 positive vessels in tumor center and tumor periphery expressed as CD34 positive vessels /mm^2^. Black bars = tumor periphery, Grey bars = tumor center. Significantly more CD34 positive vessels are seen in the tumor center (*P<0.001). Quantification of αSMA pericyte association with tumor vessels revealed a significantly greater number in the tumor periphery compared to the tumor center (* P<0.0001, Black bars = tumor periphery, Grey bars = tumor center. Data is expressed as mean value± SEM, with n≥5 for each group. Data was not normally distributed and non-parametric analysis was performed and statistical significance determined using Kruskal Wallis and Mann-Whitney U test.

CD34 and CD31 are two endothelial cell markers often used in determining tumor vascular density. While these two markers roughly stain the same number of tumor vessels (Additional file 3: Figure S2) neither marker stains all the tumor vessels. In our experience CD34 normally stains tumor vessels and host vessels undergoing neovascularisation as seen in liver regeneration (unpublished result) but stains mature vessels only minimally. CD31 shows more cross-reactivity and also stains liver sinusoids (Additional file 3: Figure S2), therefore in this study we used CD34. Staining and quantification of CD34 positive staining vessels (Figure 1A and B) demonstrate significantly stronger staining (Figure 1A inset 1 arrows) and greater density in the central regions of tumor (Figure 1B, P<0.001). Vessels in the periphery either did not stain or only partially stained with CD34 (Figure 1A inset 2 arrows). Interestingly CD34 negative or faintly stained host vessels at the tumor-host interface were seen to be co-opted by tumor cells (Figure 1A inset 3 arrows). Maturity of tumor vessels was assessed by αSMA staining of pericytes associated with the vessels. In addition to pericytes αSMA also stains myofibroblasts and in this model there is significant accumulation of myofibroblasts within the tumor stroma, especially in the periphery (Additional file 4: Figure S3). To determine pericyte coverage we used serial sections stained for CD34 /αSMA and only vessels that stained for both markers regardless of the strength of CD34 staining were included in the enumeration. Tumor periphery showed at least 2.5 times greater pericyte coverage than vessels in the center of the tumor, indicating that the vasculature is more stable and mature in the periphery (Additional file 4: Figure S3 and Figure 1C, P<0.001). Due to the large number of αSMA staining myofibroblasts the difference in vessel pericyte coverage is only an estimate and may be underestimated since not all vessels in the periphery stain for CD34. Angiopoetin 1 another vessel stability marker was also preferentially associated with vessels in the tumor periphery (Additional file 4: Figure S3) further supporting our finding that the periphery of tumors is associated with relatively mature stable vessels.

### Spatial differences in the accumulation of immune cells

**Figure 2:**
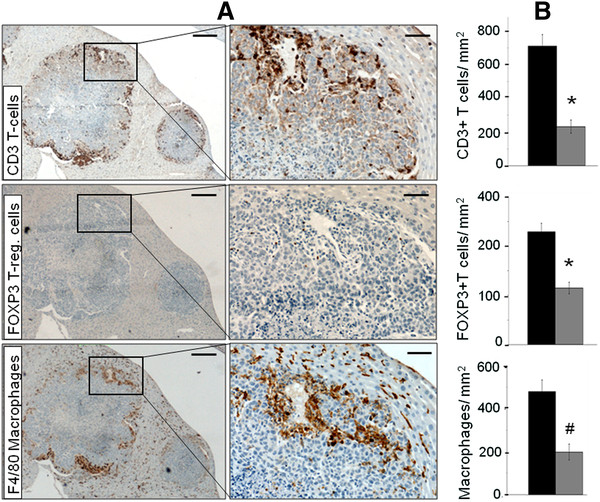
**Preferential accumulation of immune cells in the tumor periphery.** Formalin fixed liver sections with CRC liver metastases were stained with anti-CD3, anti-FOXP3 and F4/80 monoclonal antibodies to detect the presence of T cells, regulatory T cells and macrophages respectively. Low magnification scale bar=250μm, high magnification scale bar=50μm. Quantification of each cell type revealed significant differences between the tumor center and the periphery. Data is expressed as mean value of positive cells/ mm^2^±SEM with n≥5 for each group. (*P=0.0001 for T cells and regulatory T cells and #P=0.021 for macrophages).

In a previous study we reported the accumulation of immune cells within the tumor [10]. In the present study we demonstrate that accumulation of CD3 T cells, regulatory T cells and macrophages is significantly higher in the periphery than in central regions of the tumor (Figure 2A and 2B, P values 0.0001, 0.0001 and 0.027 respectively). Of particular interest was that regulatory T cells represent a significant fraction (32.4% in the periphery and 49.5% in the center) of the T cell population indicating an immunosuppressive function.

### The periphery of the tumor is normoxic relative to the center

**Figure 3:**
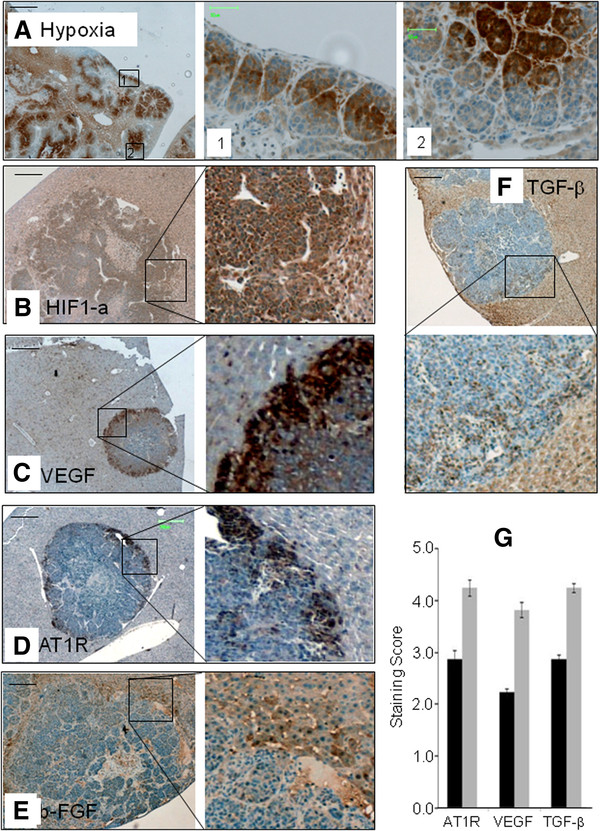
**Molecular and morphological differences between tumor periphery and center.** Formalin fixed liver sections with CRC liver metastases were stained for hypoxia by staining for pimonidazole using hypoxiprobe and growth factor/receptor expression HIF1a, VEGF, AT1R , b-FGF and TGF-β using the respective antibodies. (**A**) Low magnification scale bar=500μm, inset magnification scale bar=50μm, Tumors in the periphery show less hypoxia (first row panel 1 and inset 2) unless the tumor periphery lies on the liver surface with no adjacent host tissue (inset 1). (**B**) HIF1a staining displaying higher staining towards the tumor center in areas associated with high hypoxia. (**C**) VEGF, (**D**) AT1R, (**E**) b-FGF and (**F**) TGF-β, all are expressed at higher levels in the tumor periphery. (B-F magnification scale bar=200μm). (**G**) Quantification of AT1R, VEGF, TGF-β, demonstrate significantly higher staining in the periphery (p<0. 0001, p<0. 001, and p<0.0001 respectively).

Hypoxia in tumors has been implicated in the development of resistance to therapy. In this study the distribution of hypoxic regions within the tumor were variable and occurred throughout the center. Importantly, tumor cells in the periphery were minimally hypoxic. Very few cells in the periphery were stained with pimonidazole (Figure 3A first panel and inset 2) except when tumors were growing on the liver surface. Peripheral tumor regions that did not lay adjacent to liver parenchyma or host vessels displayed hypoxia, as seen in Figure 3A (first panel and inset 1). More centrally located tumor cells, particularly those not in close proximity to major vessels, displayed high levels of hypoxia (Figure 3A). These results support our observations that the periphery of the tumor is supplied with mature and stable vessels. HIF1α expression was variable as seen with hypoxia and displayed a similar distribution pattern, indicating that it is stabilized by hypoxia, however expression is also seen in the periphery albeit at lower levels (Figure 3B).

### The tumor periphery is associated with upregulated growth factor expression

Hypoxia and HIF1α are known to stimulate up-regulation of pro-angiogenic growth factors [22]. Expression of VEGF and the pro-angiogenic receptor AT1R are markedly up-regulated in the periphery (Figure 3C and Figure 3D). However, the distribution of VEGF and AT1R were found to closely mirror the distribution of infiltrating T cells and macrophages (Figure 2A) rather than the distribution of hypoxia and HIF1α (Figure 3A and Figure 3B). This suggests that these factors may be mainly expressed by or under the influence of the infiltrating immune cells. Similarly b-FGF and TGF-β are preferentially expressed in the periphery. Additionally these two factors are also strongly expressed within the liver parenchyma immediately adjacent to the tumor host interface (Figure 3E and Figure 3F).

### The tumor periphery is associated with increased mesenchymal marker expression

**Figure 4:**
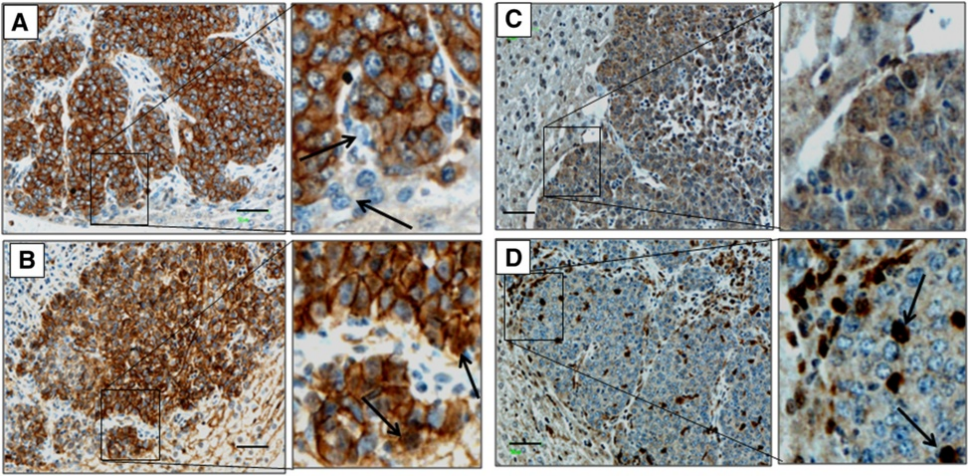
**Tumor cells in the periphery express mesenchymal markers.** Formalin fixed liver sections with CRC liver metastases were stained with antibodies for EMT markers; E-cadherin (**A**), β-catenin (**B**), vimentin (**C**) and ZEB1 (**D**). Magnification scale bar=200μm. Arrows in A magnified inset indicates detached tumor cells not expressing E-cadherin. Arrows in B magnified inset indicates tumor cells displaying nuclear localization of β-catenin. Magnified inset in C indicates increased vimentin staining in the periphery. Magnified inset and arrows in D indicate tumor cells displaying nuclear localization of ZEB1.

The bulk of the tumor cells in CRCLM were found to be strongly positive for E-cadherin and displayed the characteristic cobblestone junctional complex staining (Figure 4A). However, in the periphery a few tumor cells did not express E-cadherin and appeared detached from the main tumor (Figure 4A; inset arrows indicate E-cadherin negative tumor cells). Immunostaining showed that most tumor cells displayed a β-catenin staining pattern similar to that of E-cadherin (Figure 4B) being present mainly in the cell junctions. In the periphery however the occasional tumor cell was positive for nuclear β-catenin (Figure 4B; inset arrows point at β-catenin nuclear localization).

Tumors in this study also showed very faint cytoplasmic vimentin staining in the bulk of the tumor. Vimentin staining was slightly more intense in the periphery where the occasional tumor cell also displayed nuclear staining (Figure 4C and inset, arrows indicate nuclear vimentin).

The majority of tumor cells in our CRCLM tumor model did not express ZEB1. In contrast, strong ZEB1 staining was seen to be associated with infiltrating stromal cells that had a mainly fibroblast appearance and accumulated in the tumor-host interface and along major vessels (Figure 4D). Some of the positive cells had a round appearance and from their observed location, they may be mast cells. A few ZEB1 positive tumor cells were also present mainly in the periphery but some also interspersed throughout the tumor (Figure 4D arrows in inset pointing at positive tumor cells). Taken together, these results indicate that a proportion of tumor cells in the periphery in this tumor adopt mesenchymal morphology.

### Treatment with Oxi4503 results in endothelial cell apoptosis and rapid occlusion of tumor vessels

**Figure 5:**
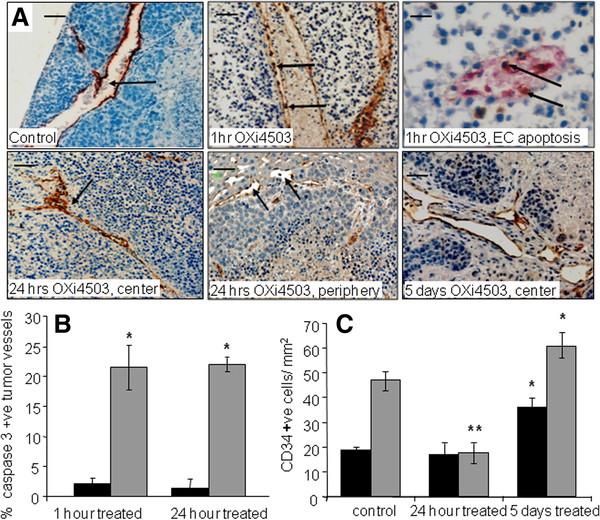
**Changes in endothelial cells and vessel morphology following OXi4503 treatment.** Mice were treated with a single IP dose of OXi4503 (100mg/kg) at 16 days after tumor induction. Tissues were collected at one hour, 24 hours and five days after treatment. Formalin fixed liver sections were stained with anti-CD34 antibody to visualize tumor vessels. (**A**), Control tumor, arrow indicates a patent tumor vessel; 1hr OXi4503, arrows indicate endothelial cells rounding up and detaching from the vessel basement membrane; 1hr OXi4503, EC apoptosis, the section was doubly immunostained for CD34 and active caspase-3 (apoptosis marker), to visualise endothelial cells undergoing apoptosis (arrows); 24hrs OXi4503 center, arrow points at a totally occluded tumor vessel; 24hrs OXi4503 periphery, arrow indicates patent tumor vessel; 5 days OXi4503 , center, demonstrating regenerating tumor vessels surrounded by proliferating tumor cells; Single staining magnification scale bar=50μm, double staining magnification scale bar=25μm. (**B**), Enumeration of vascular endothelial cell apoptosis show significant differences between the tumor center and periphery at one and 24 hours after treatment (*P <0.001); (**C**), Quantification of tumor vascular changes following OXi4503 treatment. Vascular density decreased significantly in the tumor center (**P<0.0001), but not the periphery (P=0.173) at 24 hours after treatment. Tumor revascularization at day five is significantly higher compared to the untreated control both at tumor center and the periphery (*P=0.001). Results are mean values ± SEM, (n≥5). Black bars = tumor periphery, Grey bars = tumor center.

Having demonstrated several important molecular and morphological differences between the periphery and the rest of the tumor, the differential effect of a single dose of OXi4503 on established CRLCM was then investigated. We examined microvascular changes in the tumor center and periphery at one hour, 24 hours and five days following OXi4503 treatment. Untreated tumors displayed open functional vessels (Figure 5 control). Within one hour of treatment tumor vessels became congested (Figure 5 1hr OXi4503). The endothelial cells lining the vessels appeared rounded and detached from the vessel wall (Figure 5, 1hr OXi4503 arrows indicate rounding endothelial cells). Using double staining for active caspase-3 and CD34, we found that endothelial cells not only changed shape but also were apoptotic (Figure 5, 1hr OXi4503). At 24 hours, all the central tumor vessels had occluded and the majority no longer stained with CD34 indicating endothelial cell death (Figure 5, 24hrs OXi4503, center and Additional file 5: Figure S4). However, a number of patent vessels that did not stain or only partially stained with CD34 were seen in the periphery (Figure 5, 24hrs OXi4503, periphery and Additional file 5: Figure S4). By day five, as seen in our previous studies [23] the tumor had vigorously re-grown towards the necrotic center and vessels had re-established with increased vessel density compared with control tumors (Figure 5, 5days OXi4503, center).

Quantification of vascular endothelial cell apoptosis by active caspase-3 staining demonstrated that OXi4503 induced significantly more vascular endothelial cell apoptosis in the tumor center at one and 24 hours after treatment compared to the periphery (Figure 5B, P <0.001 for both timepoints). This differential in apoptosis of vascular endothelial cells resulted in a significant decrease in vascular density in the tumor center (P<0.0001), but no significant change in the periphery (P=0.173) at 24 hours after treatment (Figure 5C).

Tumor vessel density in the treated tumor five days after treatment was found to be significantly higher compared to the untreated control both in the bulk of the tumor and in the periphery. Vascular density is 1.6 times higher in the periphery and 1.9 times higher in the center of OXi4503 treated tumors compared to controls (Figure 5C, P<0.001 for both). These results demonstrate inherent differential resistance to OXi4503 in tumor vasculature between the periphery and the bulk of the tumor. Furthermore after the initial vessel damage revascularization resumed at increased rates indicating that treatment induced signals for angiogenesis.

### Tumor cells in the periphery are resistant to apoptosis after OXi4503 treatment

**Figure 6:**
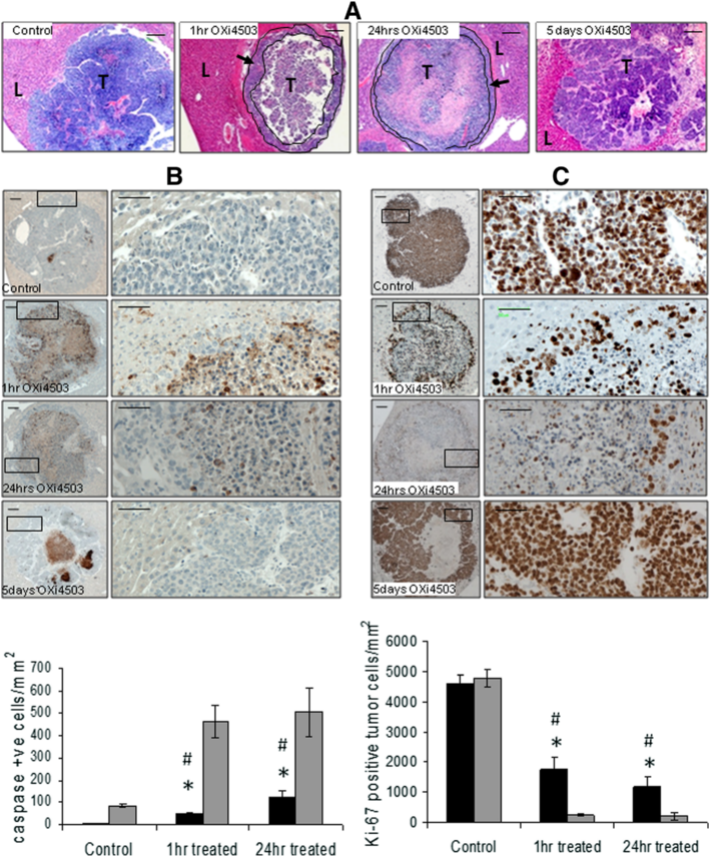
**Changes in tumor kinetics following OXi4503 treatment.** Mice were treated with a single IP dose of OXi4503 (100mg/kg) at 16 days after tumor induction. Tissues were collected at one hour, 24 hours and five days following OXi4503 treatment. (**A**). H&E stained sections at indicated times after OXi4503 treatment. Magnification scale bar=200μm. Tumor cells at the live rim at one and 24hrs are seen in the enclosed lined areas indicated by arrows. NA= necrotic/apoptotic area, T= tumor, L=liver. (**B**) Tumor cell apoptosis at indicated times after treatment detected by active caspase-3 staining. Low magnification scale bar=250μm, inset magnification scale bar=50μm. Graph showing quantification of apoptotic tumor cells. Results are mean values ± SEM, (n≥5). Black bars = tumor periphery, Grey bars = tumor center. Apoptosis in the treated tumor periphery was significantly higher than in the periphery of control tumors at one and 24 hours (*P <0.001) but significantly lower than the center of the treated tumors (#P<0.0001). (**C**) Proliferation changes in tumors at indicated times after treatment detected by Ki-67 staining. Low magnification scale bar=250μm, inset magnification scale bar=50μm. Graph showing quantification of Ki-67 positive tumor cells. Results are mean values ± SEM, (n≥5). Black bars = tumor periphery, Grey bars = tumor center. Proliferation in the periphery was significantly reduced at one and 24 hours (*P <0.001) following treatment compared to controls. Significantly higher number of cells proliferate in the periphery of treated tumors compared to the center (#P<0.0001).

Following OXi4503 treatment (Figure 6A) a thin rim of viable cells was seen in H&E stained tumor sections at the tumor host interface at both one hour and 24 hour timepoints. No appreciable change in the number of viable tumor cells could be seen between these two timepoints. In control tumors, apoptosis occurs within some central regions but very seldom within the periphery (Figure 6B control). Within one hour following treatment, significant apoptosis occurred in the tumor center leading to large necrotic areas (Figure 6B, 1hr OXi4503). This pattern of injury continued at 24 hours (Figure 6B 24 hrs OXi4503). Although apoptosis in the periphery was significantly increased at one and 24 hours after OXi4503 compared to controls (Figure 6B graph, P<0.0001 for both timepoints) there was significantly lower apoptosis in the treated periphery compared to center of the treated tumor (Figure 6B graph, P<0.0001 for both timepoints). Furthermore, significantly more apoptotic cells are seen in the periphery at 24 hours compared to one hour after treatment, suggesting that inhibition of apoptotic pathways immediately following treatment may be part of the resistance mechanism. By five days after treatment apoptosis had virtually ceased and new tumor growth is seen to extend towards the center into previously apoptotic areas (Figure 6A and Figure 6B, 5 days OXi4503).

### Tumor cells in the periphery transiently reduce proliferation after OXi4503 treatment

Quantification of tumor cell proliferation showed that control tumors exhibited high rates of proliferation in both the center and the periphery (Figure 6C control). Cell proliferation was drastically reduced both at the center and the periphery at one and 24 hours after treatment (Figure 6C, 1 hr and 24 hrs, P<0.001 in all cases compared to untreated control). Comparison of tumor proliferation between periphery and center in treated tumors showed that significantly higher cell proliferation was seen in the periphery at both timepoints (Figure 6C, 1 hr and 24 hrs. P<0.0001 for both timepoints) These results show that a significant proportion of tumor cells in the periphery stop proliferating in response to VDA treatment, reaching a minimum at 24 hours after treatment. Reduction in proliferation was seen to be only a transient response however, as by day 5 these cells have recovered and resumed vigorous proliferation (Figure 6A, 5days OXi4503 and 6C, 5days OXi4503).

## Discussion

Tumor microvasculature unlike that of the host is particularly sensitive to vascular disruptive agents such as OXi4503 resulting in rapid vessel thrombosis and significant tumor cell death. A single dose of OXi4503 in mice at the MTD produces more than 90% necrosis of the total tumor mass. Characteristically complete tumor eradication is not achieved as a thin rim of viable tumor in the periphery invariably gives rise to regrowth [6,7,16].

The first part of this study demonstrates several inherent differences between the tumor center and periphery that may account for the differential resistance to VDA treatments.

Significantly more tumor vessels stained positive for CD34 in the center of the tumor compared to the periphery. Tumor vessels in the periphery display greater pericyte coverage and angiopoetin 1 association than vessels in the tumor center. In our experience CD34 only stains tumor vessels and vessels undergoing neoangiogenesis as seen in liver regeneration (unpublished data). Our results indicate an inverse relationship between CD34 and αSMA staining. The presence of αSMA expressing myofibroblasts in our tumor model may have resulted in an underestimation of the difference in vessel maturity between tumor center and periphery. The lower expression of CD34, greater presence of pericytes and angiopoietin 1 association in the periphery suggests significantly greater maturation of the microvasculature in that region [18]. These findings suggest vessels in the center of the tumor are under constant remodeling while periphery is supplied by more mature and stable vessels. Other differences between the center and the periphery also include lower levels of hypoxia in the periphery and significantly higher expression of proangiogenic factors and receptors (VEGF, TGF-β, b-FGF and AT1R). The relatively stable mature vessels in the periphery and close proximity to normal host vessels are likely the reason for minimal hypoxia However, in contrast to current opinion increased proangiogenic factor expression, with the exception of HIF1α, does not overlap with regions of increased hypoxia in our study [22]. Instead we observed a close overlap of increased proangiogenic factor expression and infiltrating immune cells (macrophages and T-cells). Other studies have also reported accumulation of infiltrating cells including macrophages and T-cells in the periphery of tumors expressing growth factors and cytokines that are proangiogenic and cytoprotective to tumor [11-14]. This phenomenon has been noted in both surgically removed human tumors and in experimental tumor models including CRC. Pro-angiogenic growth factors such as VEGF in addition to their role in neovascular formation are also directly cytoprotective to cells expressing their receptors including endothelial and tumor cells [24,25]. In addition to the growth factors and cytokines we investigated in this study, there are several other studies reporting additional pro-tumor cytokines, enzymes and growth factors being up regulated in the periphery of the tumor [26-31]. The tumor cells at the host interface are morphologically different and are reported to have undergone EMT, perhaps as a result of the higher growth factor influence, conferring on them characteristics such as increased invasive ability and drug resistance [20,21,32-34]. In our study mesenchymal markers ZEB1 and vimentin were preferentially expressed in the tumor periphery, while the epithelial markers E-cadherin and β-catenin were reduced from cell junctions of some cells in the periphery, suggesting that these cells have undergone EMT. Our findings in the first part of this study therefore demonstrate that the tumor microenvironment in the periphery is significantly different to that of the rest of the tumor and may account for the differential response to OXi4503 treatment.

The second part of our study investigated the effect of OXi4503 treatment on tumor microvasculature and tumor cell kinetics. We demonstrated that vessels in the periphery are resistant to OXi4503 as they remain patent following treatment. This resistance correlates with the increased vessel maturity and stability in the periphery and spatially overlaps with the observed immune cell accumulation and increased growth factor expression seen in control tumors. Initially it was assumed that tumor cells in the periphery survive VDA treatment due to their close proximity to host vessels [2,35] but recent studies demonstrated retained perfusion within the viable rim and patent vessels in the periphery as also seen in our study [3,7,36,37]. However, to our knowledge this study is the first to demonstrate and correlate the maturity of the microvasculature in the periphery to its ability to resist the effects of VDAs. A clinical study by Gaya et al. [38] investigating the effect OXi4503 treatment on a variety of different tumors reported significant increase in vessel permeability correlated with high expression of angiopoetin 2, a marker of vessel instability [18]. While that work involved observations on whole tumor, the result supports our finding that vessel stability correlates with VDA resistance. Different types of tumor differ in the degree of vascularization, in their vessel morphology and maturity. This variation likely influences the effectiveness of OXi4503 treatment. Wankhede et al. [37] showed a mouse mammary carcinoma (4T1) and a human renal cell carcinoma (Caki-1) xenograft were differentially resistant to OXi4503 treatment when grown in mouse dorsal window chambers. They speculated differences in microenvironment may account for the observation. While the tumor periphery does not fully succumb to the effects of VDA treatments, our study and others have demonstrated that some vessels in the periphery are affected [36,37]. Other studies also reported some decrease in perfusion within the viable rim and indications of increased hypoxia [7,36,37]. Hypoxia is known to inhibit proliferation and indeed our results show significantly reduced proliferation in the periphery after treatment. Reduced proliferation was also reported following VDA treatments even when apoptosis was not seen [36,37]. We demonstrated that both apoptosis and proliferation of tumor cells are differentially modulated in the periphery following OXi4503 treatment. Evasion of apoptosis and temporary inhibition in proliferation are mechanisms adopted in drug resistance [39]. Cells with mesenchymal characteristics have migratory properties and do not proliferate. It is possible that the tumor cells within the periphery are protected by their specific microenvironment, but the stress of the treatment and the ensuing hypoxia may transiently push them further in the direction of mesenchymal morphology so they temporarily cease proliferation.

Metastasis is responsible for over 90% of cancer deaths and in colorectal cancer it accounts for more than 70% of mortality [40]. The majority of systemic therapies for cancer including chemotherapy and biologically targeted therapies appear to achieve partial tumor response with varying amounts of residual tumor cells surviving treatment. Even if initial tumor regression is achieved, the surviving tumors have been shown to develop resistance to chemotherapy and behave with increased invasiveness. One explanation for this resistance is the accumulation of mutations in the constantly proliferating tumor cells enabling the selection of aggressive resistant clones [41]. While this may be partly responsible for tumor recurrence, the role of the tumor microenvironment and the host stromal cells in drug resistance has been overlooked until recently. Resistance has also been attributed to vascular inefficiency resulting in failure to deliver adequate drug concentration into the center of the tumor leading to incomplete destruction [42]. In other published studies using VDAs including our own work, this has not been a problem since all surviving tumor is associated with the periphery [3,6,43]. Recently it has been noted that tumors are heterogeneous and not all cells are equally capable of giving rise to metastasis [44]. Cytotoxic drugs kill cells that readily divide and are usually differentiated. A proportion of tumor cells are slow dividing, have a less differentiated morphology and are capable of giving rise to metastasis more efficiently. These cells have been termed cancer stem cells (CSC) and have been shown to express some progenitor stem cell characteristics and are resistant to drugs [45]. CSCs are reported to reside in perivascular niches [46] and most commonly at the tumor host interface [47,48]. In more recent studies it has been demonstrated that tumor cells in culture acquire cancer stem cell characteristics when treated with agents that promote EMT [49]. Other studies have shown that *in vivo* drug treatment of tumors leads to increased frequency of mesenchymal and stem cell phenotypes in the recurrent tumor [34,50]. Our results complement the recent literature by showing cell survival in the periphery coinciding with the suggested niche of stem cells. It is not entirely clear if the cells in the periphery survive because they are stem cells or because they have a better vascular support and a milieu of protective cytokines. It is possibly due to a combination of these factors. Future studies on molecular changes on the surviving cells after treatment, in terms of stem cell marker expression and EMT state will shed more light on the mechanisms that protect these cells from apoptosis.

## Conclusion

In summary this study has identified a number of morphological and molecular differences between the bulk of the tumor and the periphery that may account for the resistance to VDAs that is specifically associated with the periphery. A better characterization of the tumor cells in the periphery before and after treatment could lead to rational drug combination therapies for total tumor eradication.

## Competing interests

The authors declare that they have no competing interests.

## Authors’ contributions

LN carried out the majority of the experiments collected data, contributed to data analysis and to manuscript draft. TF contributed in the experimental design, assisted in experimental work and data analysis and wrote the manuscript. MC-W assisted in experimental work, data analysis and statistics and edited the manuscript. LC performed some of the animal experiments and the immunostaining for hypoxia, b-FGF and TGF-β. PLC assisted with data analysis and statistics. MN, VM and CC contributed to study design and edited the manuscript. CC is the head of the department. All authors have read and approved the final manuscript.

## Pre-publication history

The pre-publication history for this paper can be accessed here:

http://www.biomedcentral.com/1471-2407/12/522/prepub

## Supplementary Material

Additional file 1 **Figure S1.** MoCR Liver metastases. Metastases are induced by intrasplenic injection of 5x 10^4^ tumor cells. (A) Liver with metastases at 18 days post tumor induction. (B) Liver slices with metastases at 21 days post tumor induction, as used to calculate tumor load. (C) H&E stained liver section containing metastases of varying sizes. Click here for file

Additional file 2 **Table S1.**List of antibodies and conditions used. Click here for file

Additional file 3 **Figure S2.** Tumor vascular staining with CD31 and CD34 endothelial cell markers. Sections of the same MoCR tumor were stained. Low magnification scale bar=250 µm, high magnification scale bar=50µm. A and C: CD31 staining detected with DAB (brown), B and D: CD34 staining detected with Vulcan fast red. Both markers stain approximately equal number of tumor vessels, in addition CD31 stains liver vessels and sinusoids. Click here for file

Additional file 4 **Figure S3.** Spatial differences in tumor vessel maturity in solid tumors. (A), Formalin fixed liver sections with CRC liver metastases. Low magnification scale bar=500 µm, high magnification scale bar=25 µm. (1-4), stained with antibodies to aSMA (staining of pericytes on mature vessels) detected with DAB (brown). (5-6), stained with antibodies to CD34 endothelial cell marker detected with Vulcan fast red. Image1, depicts a low magnification of a whole tumor section. Images 2 and 3 depict host vessels and tumor vessels respectively in the periphery staining positive for pericytes. Image 4 depicts a central tumor vessel staining negative for pericytes. Images 5 and 6 are sections from the same tumor showing strong CD34 staining of the central vessel while the peripheral vessels show only weak and partial staining. Tumor fibroblasts and some tumor cells are also positive for αSMA. Pericytes are mostly flat cells lining the vessels (arrows). (B) Double staining for Angiopoetin 1(vessel maturity marker) detected with DAB (brown), and CD34 endothelial cell marker detected with Vulcan fast red. Scale bar=250 µm. Angiopoetin1 is preferentially associated with the periphery as shown in inset 1and inset 2 (a peripheral vessel between two adjacent tumors and a central vessel respectively). Click here for file

Additional file 5 **Figure S4.** Endothelial cell and tumor cell apoptosis following OXi4503 treatment. Low magnification scale bar=250 µm, high magnification scale bar=50 µm. Formalin fixed liver sections with CRC liver metastases (A) control and (B) 24 hours following OXi4503 treatment. Inset 1 shows tumor periphery and inset 2 shows tumor center. Sections were doubly immunostained for CD34 and active caspase-3 (apoptosis marker) and detected with Vulcan fast red and DAB (brown) respectively. Control tumor shows some areas of tumor cell apoptosis but no double staining is apparent. In contrast treated tumors show extensive double staining indicating vascular endothelial cell apoptosis as indicated with black arrows. Red arrows in inset B1 indicate patent vessels in the tumor periphery. Click here for file
