# Supplementary figures and images for: Spatial morphological and molecular differences within solid tumors may contribute to the failure of vascular disruptive agent treatments
Source: BMC Cancer. 2012 Nov 15;12:522. doi: 10.1186/1471-2407-12-522 (PMC3583184; doi:10.1186/1471-2407-12-522)

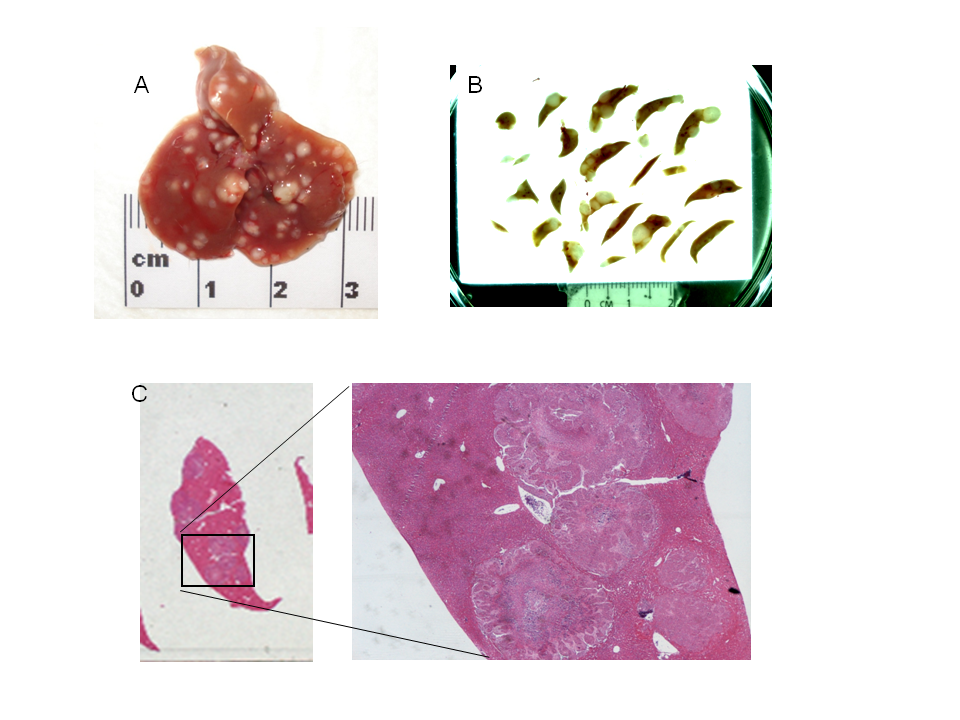

Supplement: Additional file 1 — Figure S1. MoCR Liver metastases. Metastases are induced by intrasplenic injection of 5x 10^4^ tumor cells. (A) Liver with metastases at 18 days post tumor induction. (B) Liver slices with metastases at 21 days post tumor induction, as used to calculate tumor load. (C) H&E stained liver section containing metastases of varying sizes. [file 1471-2407-12-522-S1.tiff]

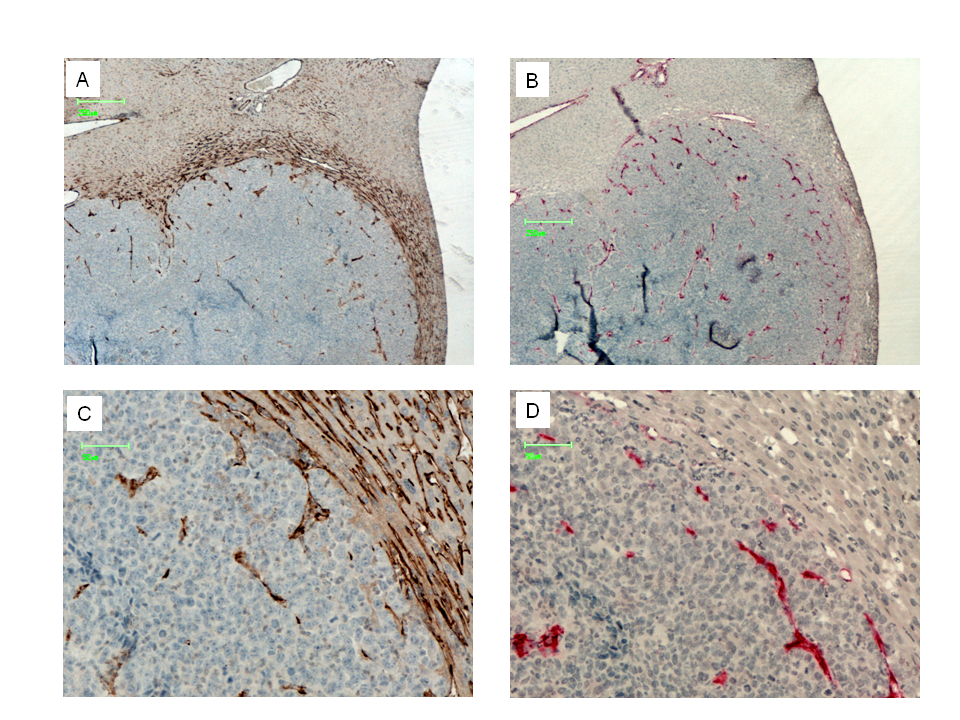

Supplement: Additional file 3 — Figure S2. Tumor vascular staining with CD31 and CD34 endothelial cell markers. Sections of the same MoCR tumor were stained. Low magnification scale bar=250 µm, high magnification scale bar=50µm. A and C: CD31 staining detected with DAB (brown), B and D: CD34 staining detected with Vulcan fast red. Both markers stain approximately equal number of tumor vessels, in addition CD31 stains liver vessels and sinusoids. [file 1471-2407-12-522-S3.tiff]

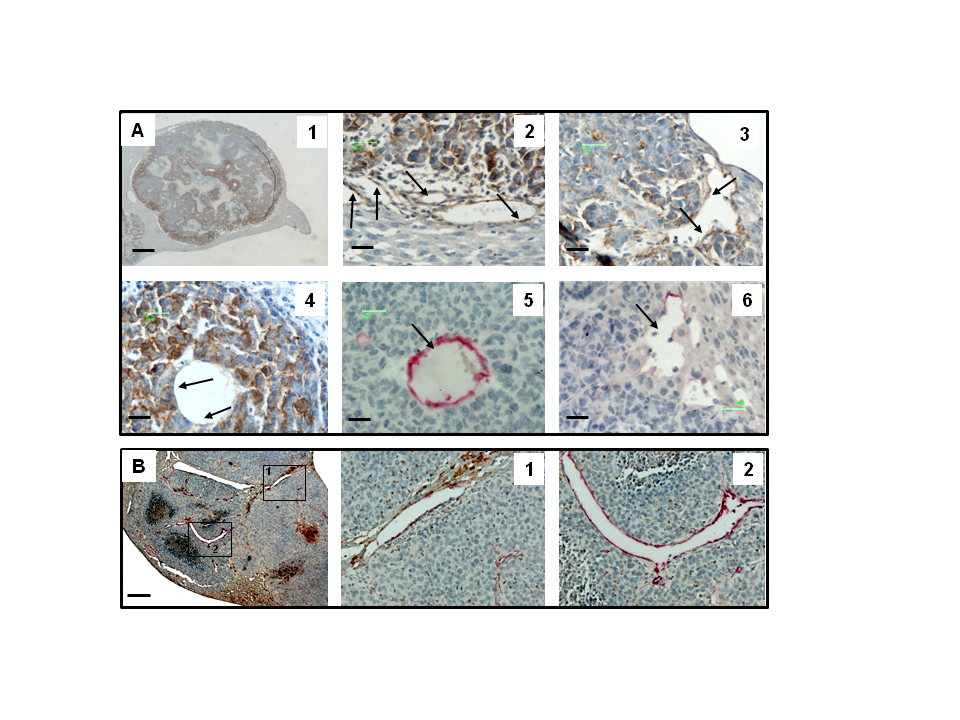

Supplement: Additional file 4 — Figure S3. Spatial differences in tumor vessel maturity in solid tumors. (A), Formalin fixed liver sections with CRC liver metastases. Low magnification scale bar=500 µm, high magnification scale bar=25 µm. (1-4), stained with antibodies to aSMA (staining of pericytes on mature vessels) detected with DAB (brown). (5-6), stained with antibodies to CD34 endothelial cell marker detected with Vulcan fast red. Image1, depicts a low magnification of a whole tumor section. Images 2 and 3 depict host vessels and tumor vessels respectively in the periphery staining positive for pericytes. Image 4 depicts a central tumor vessel staining negative for pericytes. Images 5 and 6 are sections from the same tumor showing strong CD34 staining of the central vessel while the peripheral vessels show only weak and partial staining. Tumor fibroblasts and some tumor cells are also positive for αSMA. Pericytes are mostly flat cells lining the vessels (arrows). (B) Double staining for Angiopoetin 1(vessel maturity marker) detected with DAB (brown), and CD34 endothelial cell marker detected with Vulcan fast red. Scale bar=250 µm. Angiopoetin1 is preferentially associated with the periphery as shown in inset 1and inset 2 (a peripheral vessel between two adjacent tumors and a central vessel respectively). [file 1471-2407-12-522-S4.tiff]

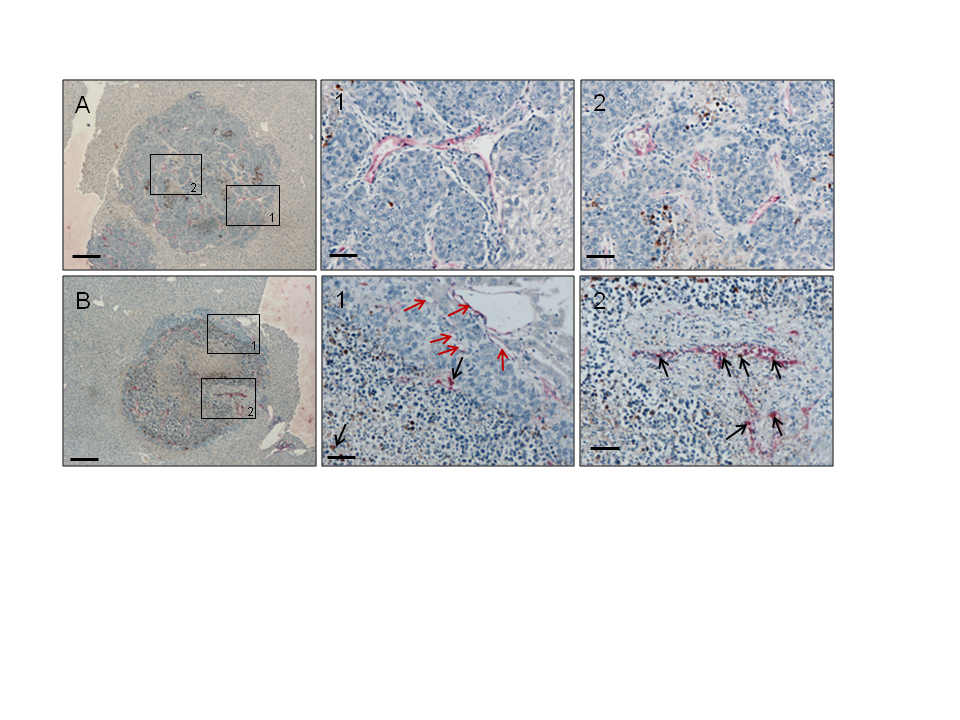

Supplement: Additional file 5 — Figure S4. Endothelial cell and tumor cell apoptosis following OXi4503 treatment. Low magnification scale bar=250 µm, high magnification scale bar=50 µm. Formalin fixed liver sections with CRC liver metastases (A) control and (B) 24 hours following OXi4503 treatment. Inset 1 shows tumor periphery and inset 2 shows tumor center. Sections were doubly immunostained for CD34 and active caspase-3 (apoptosis marker) and detected with Vulcan fast red and DAB (brown) respectively. Control tumor shows some areas of tumor cell apoptosis but no double staining is apparent. In contrast treated tumors show extensive double staining indicating vascular endothelial cell apoptosis as indicated with black arrows. Red arrows in inset B1 indicate patent vessels in the tumor periphery. [file 1471-2407-12-522-S5.tiff]
