# Supplementary material for: Spatial morphological and molecular differences within solid tumors may contribute to the failure of vascular disruptive agent treatments
Source: BMC Cancer. 2012 Nov 15;12:522. doi: 10.1186/1471-2407-12-522 (PMC3583184; doi:10.1186/1471-2407-12-522)
Supplement: Additional file 2 — Table S1.List of antibodies and conditions used. [file 1471-2407-12-522-S2.doc]

Additional file 1: **List of antibodies and conditions used.**

| **Target protein** | **Company** | **Antibody** | **Dilution** | **Conc. (μg/ml)** | **Antigen retrieval** |
| --- | --- | --- | --- | --- | --- |
| CD34 | Serotec | Rat anti-mouse monoclonal MCA18256 | 1:500 | 2 | Citrate |
| CD31 | Abcam | Rabbit anti-mouse ab 28364 | 1:200 | 1 | Citrate |
| β-catenin | Santa Cruz | Rat anti-mouse monoclonal sc-7199 | 1:300 | 0.67 | Tris |
| E-cadherin | Santa Cruz | Rabbit anti-mouse sc-7870 | 1:500 | 0.4 | Citrate |
| Vimentin | Santa Cruz | Rabbit anti-mouse sc-5568 | 1:300 | 0.67 | Tris |
| ZEB1 | Santa Cruz | Rabbit anti-mouse sc-25388 | 1:200 | 1 | Tris |
| AT1R | Santa Cruz | Rabbit anti-mouse  sc-1173 | 1:300 | 0.67 | N/A |
| Active Caspase-3 | R&D systems | Rabbit anti-mouse AF835 | 1:800 | 0.25 | Citrate |
| αSMA | Biocare | Rabbit anti-mouse CME 305 AB | 1:300 | 33.33 | None |
| VEGF | CalBiochem***,*** | Rabbit anti-mouse PC315 | 1:400 | 2.5 | Proteinase K |
| Angpt1 | Abcam | Rabbit anti-mouse ab 8451-200 | 1:1000 | 75 | Citrate |
| Ki-67 | Thermo Scientific | Rabbit anti-mouse RM-9106-S1 | 1:100 |  | DAKO retrieval |
| CD3 | DAKO | Rabbit anti-mouse A0452 | 1:1000 | 0.6 | Proteinase K |
| FOXP3 | e-bioscience | Rat anti-mouse monoclonal 14-5773-80 | 1:100 | 5 | Citrate |
| F4/80 | Produced in house | Rat anti-mouse monoclonal ascites | 1:20000 |  | Citrate |
| HIF1-α | Chemicon | Rabbit anti-mouse AB 3883 | 1:100 | 60 | Tris |
| b-FGF | Santa Cruz | Rabbit anti-mouse Lot no:24030710 |  | 4 | Citrate |
| TGF-β | Santa Cruz | Rabbit anti-mouse sc7892 |  | 2 | Citrate |
